# Role and Functional Domain of Hepatitis B Virus X Protein in Regulating HBV Transcription and Replication *in Vitro* and *in Vivo*

**DOI:** 10.3390/v5051261

**Published:** 2013-05-22

**Authors:** Dao-Yin Gong, En-Qiang Chen, Fei-Jun Huang, Xiao-Hua Leng, Xing Cheng, Hong Tang

**Affiliations:** 1 Center of Infectious Diseases, West China Hospital, Sichuan University, Chengdu 610041, China; E-Mails: daoyingong@163.com (D.-Y.G.); chenenqiang1983@hotmail.com (E.-Q.C.); xhleng99@163.com (X.-H.L.); chengxing0013@126.com (X.C.); 2 Division of Infectious Diseases, State Key Laboratory of Biotherapy, Sichuan University, Chengdu 610041, China; 3 Department of Forensic Pathology, Medical School of Basic and Forensic Sciences, Sichuan University, Chengdu 610041, China

**Keywords:** hepatitis B virus, HBx protein, transcription and replication, cell culture, mouse model

## Abstract

The role of hepatitis B virus (HBV) X protein (HBx) in the regulation of HBV replication remains controversial. In the present study, the role of HBx in regulating HBV replication was initially investigated in both HepG2 and Huh7 *in vitro* cell lines with a transient transfection system. Next, the regions of HBx responsible for transcriptional transactivation and promotion of HBV replication were mapped in an HBV replication mouse model by *in vivo* transfection of a series of HBx expression plasmids. In an *in vitro* setting, HBx deficiency had little effect on HBV replication in Huh7 cells, but impaired HBV replication in HepG2 cells. In an *in vivo* setting, HBx had a strong enhancing effect on HBV transcription and replication. For the C-terminal two-thirds of the protein (amino acids [aa] 51 to 154) was required for this function of HBx, and the regions spanning aa 52 to 72 and 88 to 154 were found to be important for the stimulatory function of HBx on HBV replication. In conclusion, the role of HBx in HBV replication regulation is affected by host cell type, and HBx has an important role in stimulating HBV transcription and replication in hepatocytes *in vivo*. Further, the transcriptional transactivation function of HBx may be crucial for its stimulatory effect on HBV transcription and replication.

## 1. Introduction

Chronic hepatitis B virus (HBV) infection is a worldwide health problem, and it has become one of the major causes of end-stage liver disease, including cirrhosis and hepatocellular carcinoma (HCC) [1,2]. In the past decade, the crucial role of HBV in hepatocarcinogenesis has been well established, but the mechanisms underlying how HBV induces malignant transformation of hepatocytes remains unclear [3]. HBV X (HBx) is a 154-amino-acid (154-aa) multifunctional protein that has roles in gene transcription, cell proliferation, and apoptosis [4,5,6]. For a long time, HBx has been suspected of playing positive roles in hepatocarcinogenesis, possibly by affecting viral replication and viral proliferation directly or indirectly [6,7,8].

In the past decade, the functions of the HBx protein in the HBV life cycle have been examined in several experimental systems [4]. In woodchucks, the HBx protein is essential for the successful establishment of woodchuck hepatitis virus (WHV) infection [9,10]. In hepatoma cell lines or HBV transgenic mice models, HBV mutant genomes with a defective X gene are replication competent after being transfected with HBx expression plasmid [11,12,13,14]. The differences observed in these experiments may suggest a critical role of the X protein in the establishment of virus infection, and not in the establishment of viral replication. However, our previous studies indeed showed that HBx could augment HBV transcription and replication, as a mutant HBV genome with a defective X gene led to decreased levels of 3.5-kb HBV RNA and HBV replication intermediates; these decreases could be restored by either transient ectopic expression of HBx or stable HBx expression in HepG2 cells [10]. Although the mechanisms underlying the involvement of HBx in HBV infection and replication are not fully elucidated, the difference in regulatory factors in different types of cells has been widely implicated [15]. Unfortunately, the body of in-depth research is still relatively small.

It has been shown that the HBx genome consists of an N-terminal negative regulatory domain and a C-terminal transactivation or coactivation domain; the C-terminus is the functional domain of HBx and can transactivate a variety of viral and cellular promoters, including the HBV promoters [11,16]. In fact, our previous study discovered that for the C-terminal two-thirds of the protein (amino acids [aa] 51 to 154) is required for this function of HBx, and the regions spanning aa 52 to 65 and aa 88 to 154 are important for the transactivation or coactivation activities of HBx and its stimulatory function in HBV transcription and replication [16]. However, all these experiments were performed in an *in vitro* system. Whether the transcriptional transactivation function of HBx is involved in regulating HBV replication *in vivo* and whether *in vivo* and *in vitro* environments have different effects on HBV replication regulation are still open questions that need to be explored.

In an attempt to fully understand the role of environmental factors on HBx protein in regulating HBV transcription and replication, we adopted two different cell culture systems *in vitro* and an immunocompetent HBV replication mouse model *in vivo*. Moreover, we sought to verify roles of the transcriptional transactivation regions in the C-terminal transactivation domain of HBx in modulating the levels of HBV transcription and replication under physiological conditions *in vivo*.

## 2. Results

### 2.1. Effect of HBx on Wild-Type HBV Transcription and Replication in HepG2 and Huh7 Cells

**Figure 1:**
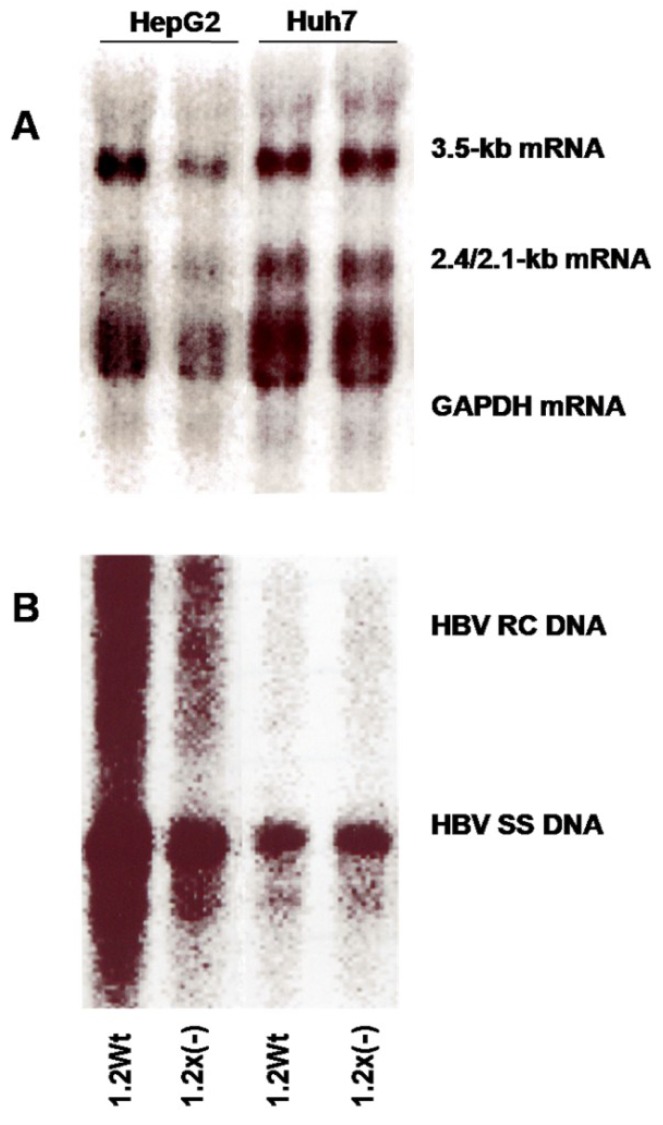
Stimulatory effects of hepatitis B virus X protein (HBx) on hepatitis B virus (HBV) transcription and replication *in vitro*. HepG2 and Hun7 cell lines were transiently transfected with the wild-type HBV construct payw1.2 (1.2wt) or the HBx-deficient HBV construct payw*7 [1.2X(−)].

The HBV RNA transcripts and replication intermediates synthesized from a wild-type HBV genome and an HBx-deficient HBV genome in HepG2 and Huh7 cell lines are shown in Figure 1. In HepG2 cells, the levels of HBV RNA transcripts and replication intermediates synthesized from the HBx-deficient HBV genome were two- to four-fold lower than were synthesized from the wild-type HBV genome. In Huh7 cells, on the other hand, the levels of both HBV RNA transcripts and replication intermediates were similar between wild-type and HBx-deficient HBV genomes. Our findings suggest that HBx can enhance HBV replication in HepG2 cells, but not in Huh7 cells. 

### 2.2. Effect of HBx on Wild-Type HBV Transcription and Replication *in Vivo*

**Figure 2:**
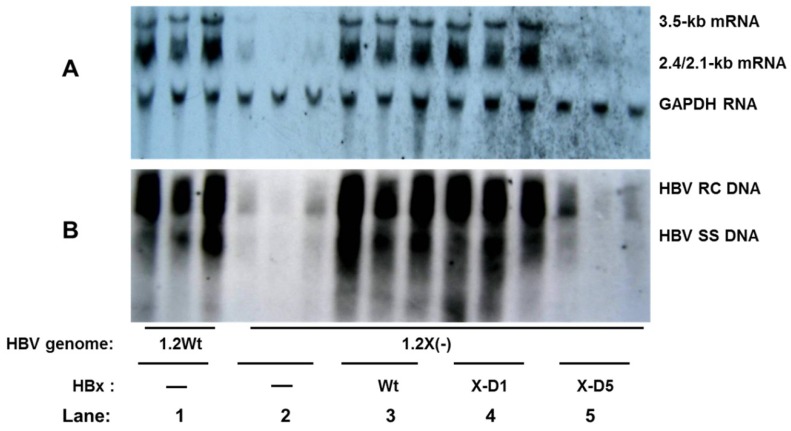
Stimulatory effects of HBx on HBV transcription and replication *in vivo*. Male BALB/c mice were introduced by hydrodynamic injection with the wild type HBV construct payw1.2 (1.2wt, lane 1) or the HBx-deficient HBV construct payw*7 [1.2X(−), lanes 2 to 5] plus empty vector control (−, lanes 2), full-length HBx expression vector (HBx-wt, lane 3), or different truncated HBx expression vectors (X-D1, lane 4; X-D5, lane 5). (**A**) RNA (Northern) filter hybridization analysis of HBV transcripts. The GAPDH transcript was used as an internal control for RNA loading per lane; (**B**) DNA (Southern) filter hybridization analysis of HBV replication intermediates: HBV RC DNA, HBV relaxed circular DNA; HBV SS DNA, HBV single-stranded DNA.

Due to the conflicting results observed in different cell lines, we further investigated the role of HBx in an *in vivo* setting. As shown in Figure 2, viral transcription and replication were detected on the third day in hydrodynamically-injected mice. Moreover, the levels of 3.5 kb HBV RNA (Figure 2A, lanes 2) and HBV replication intermediates (Figure 2B, lanes 2) synthesized from the HBx-deficient HBV genome were eight- to ten-fold lower than were synthesized from the wild-type HBV genome (Figure 2A,B, lanes 1). Further, the reduced levels of 3.5 kb HBV RNA (Figure 2A, lanes 3) and HBV replication intermediates (Figure 2B, lanes 3) were restored to wild-type levels after co-transfection of a plasmid encoding wild-type HBx.

In the present study, the levels of HBV replication intermediates and 3.5-kb HBV RNA were almost parallel in an *in vivo* setting, indicating that HBx has a stimulatory effect on both HBV transcription and replication.

### 2.3. Role of the C-terminal Transactivation Domain of HBx in HBV Transcription and Replication *in Vivo*

As shown in Figure 2, the N-terminal negative regulatory domain of HBx was not required for its enhancement of HBV transcription (Figure 2A, lanes 4) and replication (Figure 2B, lanes 4) *in vivo*, as a truncated HBx protein (HBxD1) lacking the N-terminal third of the protein (aa 1 to 50) stimulated HBV replication to an extent similar to that of the full-length HBx (Figure 2A,B, lanes 4). In contrast, this stimulatory effect was abolished when for the C-terminal two-thirds of the protein (aa 51 to 154), which contains the transactivation domain, was deleted (HBxD5) (Figure 2B,C, lanes 5). 

Thus, our results further indicate that the C-terminal transactivation domain of HBx is necessary and sufficient for its stimulatory function on HBV transcription and replication *in vivo*.

### 2.4. Role of Different HBx Regions in HBV Transcription and Replication Regulation *in Vivo*

**Figure 3:**
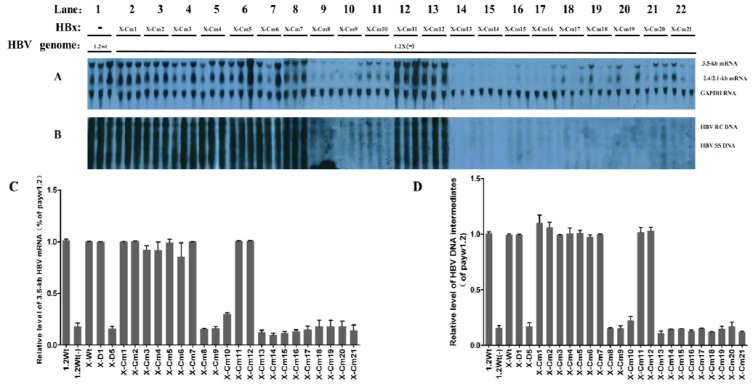
Mapping of the HBx sequences required for the stimulatory effect on HBV transcription and replication *in vivo*. Male BALB/c mice were introduced by hydrodynamic injection with the wild-type HBV construct payw1.2 (1.2wt, lane 1) or the HBx-deficient HBV construct payw*7 [1.2X(–), lanes 2–22] plus a series of clustered mutated HBx expression plasmid vectors (X-Cm1 to X-Cm21, lanes 2-22, respectively). (**A**) RNA (Northern) filter hybridization analysis of HBV transcripts. The GAPDH transcript was used as an internal control for RNA loading per lane; (**B**) DNA (Southern) filter hybridization analysis of HBV replication intermediates: HBV RC DNA, HBV relaxed circular DNA; HBV SS DNA, HBV single-stranded DNA; (**C** and **D**) Quantitative analysis of 3.5-kb HBV RNA and HBV DNA replication intermediates. The levels of 3.5-kb HBV RNA and HBV DNA replication intermediates (HBV DNA RI) are reported relative to those of the wild-type HBV construct payw1.2 plus the control vector, which were set at 1.0. The mean RNA and DNA levels plus standard deviation (indicated by error bars) of three independent analyses are shown.

As shown in Figure 3, the enhancing effects of HBx mutants Cm1 to Cm7 (aa 2 to 51) and Cm11 to Cm12 (aa 73 to 87) on HBx-deficient HBV transcription and replication were similar to that of wild-type HBx, indicating that these two HBx mutants retained the ability to complement the deficiency of HBx to a similar extent as wild-type HBx expressed *in trans*. HBx mutants Cm8 to Cm10 and Cm13 to Cm21, which span aa 52 to 72 and 88 to 154, respectively, were unable to restore the stimulatory function of HBx. As these HBx mutants cover the majority of the transactivation domain, they must be important for the enhancement function of HBx in HBV replication *in vivo*. Though the mutations in HBx mutants Cm11 to Cm12 were located between aa 73 and 87, which is within the transactivation domain, they were not necessary for the activation function of HBx on HBV replication.

## 3. Discussion

In the current study, HBx was found to be required for wild-type levels of HBV replication in HepG2 cells, but was not essential for the establishment of HBV replication in Huh7 cells. This finding indicates that the effects of HBx on HBV replication and transcription vary depending on the experimental system. This discrepancy may be due to different cell genetic backgrounds and/or environments, but the exact reasons remain unclear. In order to get a more realistic understanding of the roles of HBx in HBV transcription and replication, it is necessary to perform investigations under physiological conditions. 

By combining the HBV replication mouse model with the hydrodynamic injection method [17], we further analyzed the role of HBx in HBV transcription and replication *in vivo*. We found that HBx had a role in augmenting HBV transcription and replication; further, HBx deficiency can be complemented by ectopically expressed HBx protein. While the findings of the present study are consistent with several reports regarding the regulation of HBV DNA replication by HBx [12,18,19], they are distinct from some reported results that suggested HBx had no effect on HBV transcription [20]. In the present study, the decrease in HBV DNA replication intermediates occurred in parallel with the reduction in 3.5-kbHBV RNA; this result is also supported by recent studies [21,22] suggesting that the augmentation of HBV DNA replication by HBx is most likely the result of activation of HBV RNA transcription. Moreover, numerous reports have suggested that HBx can transactivate a variety of viral and cellular promoters and enhancers, including direct interaction with nuclear transcription components and activation of cytosolic signal transduction pathways. Thus, the increased level of viral transcription can be explained by the transactivation effect of HBx on HBV promoters. This explanation is also supported by previous *in vitro* experiments, which demonstrated that HBV C promoter activity can be trans-activated by HBx [16,23].

Though some studies have reported that HBx plays an important role in initiating and maintaining virus replication after infection [24], the stimulatory effect of HBx on HBV replication may also be important for the early steps of natural infection, as very low levels of virus may be more easily eliminated by the immune system. In this study, in a condition of HBx deficiency, the reduction in viral replication *in vivo* was more significant than observed in hepG2 cells *in vitro*. This finding is consistent with the results reported by other independent research groups. The dissimilar effects of HBx deficiency observed in *in vitro* and *in vivo* experiments may be explained by the mode of viral nucleic acid introduction. In the *in vitro* transfection experiments, a relatively large amount of HBV DNA is introduced by transfection, while *in vivo* experiment, the situation is different.

In the present study, analysis with truncated HBx proteins indicated the C-terminal transactivation domain was required for the enhancing effects of HBx on HBV transcription and replication, while the N-terminal domain was dispensable. These findings are consistent with several *in vitro* studies [25]. In previous studies, HBx was shown to act as a transcriptional coactivator but not as an activator; in the present study, this interpretation of HBx is clearly supported by our results [16], and we further demonstrated that the same sequences in the C-terminal domain were required for HBx activity in an immunocompetent HBV replication mouse model *in vivo*. Additionally, the experiments with a series of clustered alanine substitution mutants of HBx confirmed the above findings and further demonstrated that two regions within the C-terminal domain, spanning aa 52 to 72 and 88 to 154, were both critical for the stimulation of HBV transcription and replication *in vivo*. Recently, it has been reported that the HBx protein binding to damage-specific DNA binding protein 1 (DDB1) is required for HBV replication [26,27,28], and Dr. Hodgson AJ and his colleagues reported that DDB1 binding-deficient HBx point mutants [HBx(69), HBx(90/91), HBx(R96E)] failed to restore wild type levels of replication from an HBx-deficient plasmid [28]. This indicated that these three loci in HBx are important for the function of HBx in enhancing HBV replication. In fact, our findings were consistent with these reported findings, and also provided more comprehensive data of HBx in regulating HBV replication. For example, the above reported HBx point mutants [HBx(69), HBx(90/91) and HBx(R96E)] were basically the same as x-Cm10, x-Cm13, and x-Cm14 in the present study, respectively; and the results in Figure 3 also showed that HBx mutants x-Cm10, x-Cm13 and x-Cm14 mutants were all unable to restore the stimulatory function of HBx. 

In summary, findings from the present study not only fully revealed the role of HBx protein in regulation of HBV replication under different environmental factors, but also further confirmed the roles of the transcriptional transactivation regions in the C-terminal transactivation domain of HBx in modulating the levels of HBV transcription and replication under physiological conditions *in vivo*.

## 4. Experimental

### 4.1. Plasmids

Plasmid payw1.2 has been described previously, and contains 1.2 copies of the wild-type HBV genome (subtype ayw) and expresses HBV pregenomic 3.5-kb RNA under the control of the endogenous promoters of HBV [29]. The HBx-deficient mutant vector payw*7, which contains an ochre stop codon (CAA to UAA) after codon 7 (at codon 8) in the HBx ORF, was engineered by site-directed mutagenesis of payw1.2.

The mammalian expression plasmids pNKF-HBx, pNKF-HBxD1 and pNKF-HBxD5, which express full-length HBx, truncated HBx (aa 51 to 154), and truncated HBx (aa 1 to 50), respectively, have been previously described. Alanine scanning mutagenesis was employed to construct a series of clustered alanine substitution mutants (designated Cm) of HBx by site-directed mutagenesis. The mutagenesis was carried out by a splicing PCR method with mutagenic oligonucleotide primer sets. The target sequence of 7-aa residues was changed to AAASAAA, and all of the HBx-encoding DNA fragments bearing the clustered mutations were introduced into the EcoRI and BamHI sites of pNKFLAG, generating constructs pNKF-Xcm1 to pNKF-Xcm21 [16].

### 4.2. Cell Culture and Plasmid Transfection *in Vitro*

Human hepatocellular liver carcinoma cell lines HepG2 and Huh7 were used as described previously [16,30]. Transfections for viral RNA and DNA analysis were performed using 10 cm plates containing approximately 106 cells. DNA and RNA isolation were performed three days after transfection. 

### 4.3. Injection of Naked Plasmid DNA *in Vivo*

Specific pathogen-free (SPF) level male BALB/c mice (6–8 weeks old), weighing 18–20 g, were provided by Huaxi Laboratory Animal Center of Sichuan University. To determine the functional domain(s) of HBx needed for its stimulatory effect on HBV replication, viral transcription and replication were examined in hydrodynamically-injected mice with plasmids expressing full-length protein (HBx) or truncated HBx proteins lacking either the N-terminal domain (HBxD1) or the C-terminal domain (HBxD5). To further investigate the exact regions of HBx involved with regulating HBV transcription and replication *in vivo*, a series of plasmids with clustered alanine substitutions in HBx were also cotransfected.

In this study, mice were injected via the tail vein with 10 μg of HBV plasmid DNA (wild-type or HBx-deficient HBV genome) in combination with full-length HBx, or a series of deletion or mutation HBx expression plasmids (2 μg) in 2.0 mL of saline solution within 5–8 s (hydrodynamics-based *in vivo* transfection [17]); the control mice were injected with 10 μg of HBx-deficient HBV expression plasmid plus 2 μg control vector.

All mice were sacrificed three days after the injection. The serum was stored at −20 °C. The liver tissue was frozen in liquid nitrogen and stored at −70 °C prior to analysis for HBV RNA and HBV DNA replication intermediates. There were at least three mice in parallel in each group, and each experiment was carried out at least three times independently.

### 4.4. Analysis of HBV RNA and HBV DNA Replication Intermediates

Transfected cells from a single plate were divided equally and used for the isolation of HBV RNA and HBV DNA replication intermediates as described previously [31]. The frozen liver tissue was mechanically pulverized in liquid nitrogen; HBV RNA were extracted using Trizol reagent (Invitrogen; USA) according to the manufacturer’s instructions, and HBV DNA replicative intermediates were isolated from 121 μg of liver tissue powder as described previously [16,30].

In this study, RNA (Northern) and DNA (Southern) filter hybridization analysis was performed with 10 μg of total RNA and 30 μL of viral replication intermediates, respectively, as described previously. Filters were probed with digoxigenin (DIG)-labeled full-length HBV DNA to detect the HBV sequence; for RNA analysis, mouse glyceraldehydes 3-phosphate dehydrogenase (GAPDH) cDNA was used as an internal control. Filter hybridization was performed with the DIG Luminescent Detection Kit (Roche Applied Science) and X-ray film. The relative abundances of specific RNA and DNA molecules were quantitated with the Quantity One system.

## 5. Conclusions

Our data clearly indicate that the effect of HBx on HBV replication is affected by host cell type, and HBx has a stimulatory role on HBV transcription and replication. Two C-terminal regions, which reside between aa 52 to 72 and 88 to 154, were discovered to be important for the stimulatory function of HBx on HBV replication *in vivo*. Our data provide useful information and lay the groundwork for future studies of HBx biological functions and the natural process of HBV infection. 
